# Does the Sum-Frequency Generation Signal of Aromatic C–H Vibrations Reflect Molecular Orientation?

**DOI:** 10.1021/acs.jpcb.3c01225

**Published:** 2023-06-07

**Authors:** Fumiki Matsumura, Chun-Chieh Yu, Xiaoqing Yu, Kuo-Yang Chiang, Takakazu Seki, Mischa Bonn, Yuki Nagata

**Affiliations:** † Max Planck Institute for Polymer Research, Ackermannweg 10, 55128 Mainz, Germany; ‡ Graduate School of Science and Technology, Hirosaki University, Hirosaki, Aomori 036-8561, Japan

## Abstract

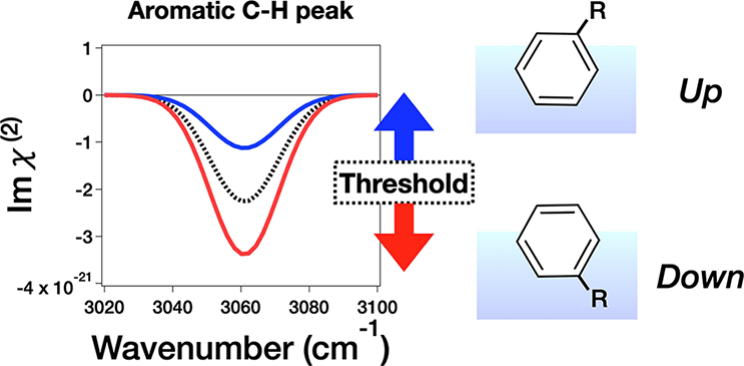

Organic molecules with aromatic groups at the aqueous interfaces play a central role in atmospheric chemistry, green chemistry, and on-water synthesis. Insights into the organization of interfacial organic molecules can be obtained using surface-specific vibrational sum-frequency generation (SFG) spectroscopy. However, the origin of the aromatic C–H stretching mode peak is unknown, prohibiting us from connecting the SFG signal to the interfacial molecular structure. Here, we explore the origin of the aromatic C–H stretching response by heterodyne-detected SFG (HD-SFG) at the liquid/vapor interface of benzene derivatives and find that, irrespective of the molecular orientation, the sign of the aromatic C–H stretching signals is negative for all the studied solvents. Together with density functional theory (DFT) calculations, we reveal that the interfacial quadrupole contribution dominates, even for the symmetry-broken benzene derivatives, although the dipole contribution is non-negligible. We propose a simple evaluation of the molecular orientation based on the aromatic C–H peak area.

## 1 Introduction

In heterogeneous catalysis, the phase of catalysts differs from that of the reactants or products.^1^ Recently, the synthesis using soft interfaces, including water/air and water/oil interfaces, has become popular because water can simplify the experimental process, provide mild reaction conditions, and sometimes deliver enhanced reactivities and selectivities.^2,3^ Such on-water synthesis is also known to accelerate the chemical reactions or increase the selectivity of the organic molecules, including benzene derivatives.^4,5^ However, information on the reaction route and structure of the reactants and intermediates at interfaces are largely lacking because probing such a chemical reaction of organic molecules at the mobile and thin interfaces has been challenging.

Heterodyne-detected sum-frequency generation (SFG) (HD-SFG) spectroscopy provides χ^(2)^ spectra, which reflect the density and orientation of molecules at interfaces. As such, HD-SFG is a powerful tool to explore the molecular level details of the chemical reaction of organic molecules owing to its surface sensitivity, molecular specificity, and orientational specificity.^6−8^ The surface sensitivity comes from the selection rule that the second-order optical response is forbidden in centrosymmetric media. The HD-SFG signal is enhanced when the IR frequency is resonant with the frequency of vibrational mode, providing molecular specificity. The sign of the Im(χ^(2)^) peak reflects the orientation of the interfacial molecules. Such a HD-SFG technique has been used for probing the interfacial organic molecules with aromatic rings. For example, by probing the aromatic C–H stretching mode with SFG, Kusaka et al. have explored the photochemical reaction of phenol^9,10^ and Seki et al. have monitored the surfactant monolayer-assisted interfacial synthesis.^11^

**Figure 1:**
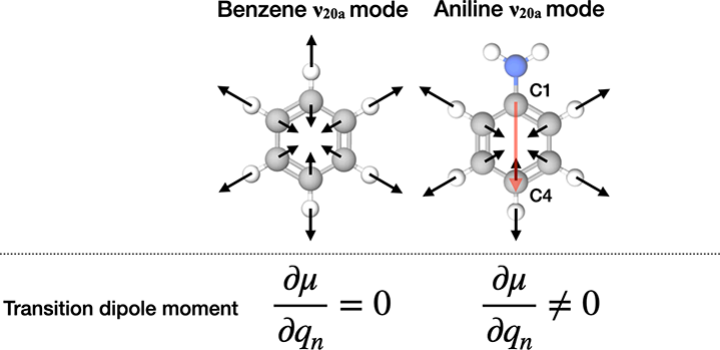
Schematic of the aromatic *v*_20*a*_ mode of benzene and aniline. Black arrows represent the directions of the motion of atoms when the molecule vibrates. The red arrow indicates the C1 → C4 direction of the aniline molecule, used as a molecular reference frame in the discussion of interfacial orientation.

If the aromatic C–H stretching Im(χ^(2)^) signal arises from the transition dipole moment, and not from the transition quadrupole moment, it provides essential information on the structure of organic molecules and biomolecules with the aromatic ring. In contrast, if a signal is dominated by the quadrupole contribution, the information on the orientation of the interfacial molecule cannot be accessed.^12−21^ The origin of the HD-SFG signal of benzene has been extensively discussed in the literature because the Im(χ^(2)^) signal has an aromatic C–H stretching peak despite benzene having no apparent transition dipole moment.^22−27^ In contrast, the origin of the aromatic C–H mode of benzene derivatives in the Im(χ^(2)^) response has not been explored, presumably because of the apparent dipole moment (see Figure 1). However, the data published so far commonly show a negative aromatic C–H stretching peak,^9,10,28−32^ questioning whether the benzene derivative aromatic C–H stretching mode reflects the molecular orientation.

To answer this question, we carried out HD-SFG measurement and density functional theory (DFT) calculation at the interfaces of several benzene derivatives: ethylbenzene, toluene, benzaldehyde, and aniline. Since these molecules are expected to have different molecular orientations at their interfaces, one can understand the dipole and quadrupole contributions to the spectra. Our HD-SFG data showed that the Im(χ^(2)^) aromatic C–H peak is all negative, irrespective of the orientations of the molecules, indicating that the quadrupole contribution is large. Yet, the Im(χ^(2)^) peak area varies substantially with the studied benzene derivatives. DFT calculations reveal that the interfacial quadrupole contribution is the largest, while the dipole contribution is non-negligible. Based on our observation, we propose a simple yet powerful method for inferring the molecular orientation of the benzene derivatives.

## 2 Theory

The second-order susceptibility χ^(2)^ can be decomposed as
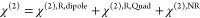
1where χ^(2), R, dipole^ and χ^(2), R, Quad^ represent the resonant contributions from the dipole term and the quadrupole term, respectively, while χ^(2), NR^ is the non-resonant contribution. In this study, we limit our discussion to the χ^(2)^ spectrum at the *YYZ* polarization direction (χ_*YYZ*_^(2)^), where the *YX*- and *XZ*-planes form the surface and the incident plane of the beams, respectively. We focus on χ_*YYZ*_^(2)^ because this polarization combination is the most frequently used beam configuration in SFG spectroscopy.^6,7,33^ The χ^(2), R, dipole^ contribution can be calculated via^12^
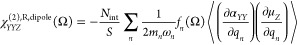
2where Ω denotes the frequency of the IR beam, *N*_int_ is the number of molecules at the interface, and *S* is the surface area. *m_n_*, ω_*n*_, *f_n_*, and *q_n_* are the reduced mass, the resonant frequency, the lineshape function, and the normal mode coordinate for vibrational mode *n*, respectively. μ and α represent the molecular dipole moment and the polarizability, respectively.

**Figure 2:**
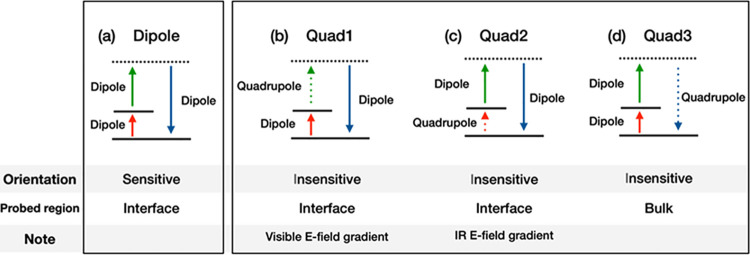
Energy diagrams for (a) dipolar mechanism and (b–d) quadrupolar mechanisms. Solid and dotted arrows represent the dipole and quadrupole transition, respectively. The information of the molecular orientation and probed region is written under the diagrams.

The χ^(2), R, Quad^ contribution arises from the three different quadrupole contributions:^26^
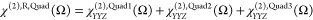
3where χ_*YYZ*_^(2), Quad1^, χ_*YYZ*_^(2), Quad2^, and χ_*YYZ*_^(2), Quad3^ represent the contributions when one of the three transitions is replaced with a quadrupole transition (see Figure 2). The Quad2 contribution arises from the molecular response at the interfaces, and its signal amplitude is proportional to the gradient of the electric field of the IR beam with respect to the surface normal (*Z* axis), while the Quad3 contribution originates from the molecules in the bulk region.^20,21^ On the other hand, χ_*YYZ*_^(2), Quad1^ = 0, because the Quad1 contribution is proportional to the gradient of electric field of the visible beam and thus is zero in the *YYZ* polarization combination. We did not consider the quadrupole contribution which arises from the gradient of the electric field in the bulk region since it has been reported to be negligible in the *YYZ* polarization combination with reflection geometry.^14,34^ The χ_*YYZ*_^(2), Quad2^ and χ_*YYZ*_^(2), Quad3^ terms are further given as^35^
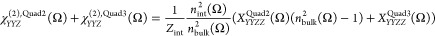
4
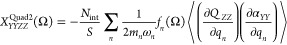
5
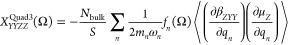
6where *n*_bulk_(Ω) and *n*_int_(Ω) denote the refractive indexes of the bulk media and interface at Ω, respectively. *Z*_int_ is the thickness of the interface, and *N*_bulk_ is the number of molecules in the bulk region. *Q* and β represent the molecular quadrupole moment and quadrupolar polarizability, respectively.

## 3 Materials and Methods

### 3.1 HD-SFG Measurement

A Ti:sapphire regenerative amplifier (Spitfire Ace, Spectra Physics) was used for the light source. A part of the output was guided to a pulse shaper consisting of a grating cylindrical mirror system to generate a narrowband visible pulse (∼15 cm^–1^). Another part of the output was converted to a broadband mid-IR pulse with an optical parametric amplifier (Light Conversion TOPAS-C, Spectra Physics) and a silver gallium disulfide (AgGaS_2_) crystal. The visible and IR beams were collinearly focused onto a 20 μm thick *y*-cut quartz to generate a local oscillator (LO) signal. A 2 mm thick SrTiO_3_ plate was inserted into the beam path to generate a time delay between LO signal and other beams. After that, visible and IR beams were again focused onto the sample surface at angles of incidence of 45°. The SFG signal from the sample surface and signal from LO were dispersed in a spectrometer and detected by a liquid nitrogen-cooled charged coupled device (CCD) camera. The SFG signals from the sample and LO interfered and generated an SFG interferogram. The complex-χ^(2)^ was obtained by Fourier analysis of the SFG interferogram and normalization by that of *z*-cut quartz crystal.^36^ The measurements were performed with *ssp* (denoting *s*-polarized SFG, *s*-polarized visible, and *p*-polarized IR beams) polarization combination.

We removed the Fresnel factor from the experimental χ_*ssp*_^(2)^ spectra to obtain χ_*YYZ*_^(2)^ spectra.^8^ We calculated the interfacial dielectric constant using the fully solvated (Lorentz) model where the interfacial dielectric constant is the same as that in the bulk.^37^ We obtained the refractive indexes of benzene derivatives from literature^38−41^ as summarized in Table S1.

For the samples, we used ethylbenzene (Tokyo Chemical Industry, purity >99.0%), toluene (Sigma-Aldrich, purity >99.7%), benzyl aldehyde (ACROS Organics, purity >99.5%), aniline (Sigma-Aldrich, purity >99.0%), and fluorobenzene (ACROS Organics, purity >99%) without further purification.

### 3.2 Computational Procedures

To estimate the dipole and quadrupole contributions for the studied molecules, we performed DFT calculations with the ORCA program package.^42^ The calculation was done at the CAM-B3LYP^43^/aug-cc-pVTZ^44,45^ level of theory. We set the thickness of the interfacial region *Z*_int_ to 6 Å by assuming that the axis profiles of the density along the surface normal for these liquids are similar to that for benzene.^23^ We assumed the Gaussian shape for the imaginary part of the lineshape function *f_n_*. The width for Gaussian function was obtained through the fit of the experimental Im(χ_*YYZ*_^(2)^) peak for each molecule with the Gaussian function. The refractive indexes of the bulk media *n*_bulk_ and the interface *n*_int_ are approximated to be constant in the IR frequency region. Furthermore, the average angles between the direction from the C1 position to the C4 position of the aromatic group (C1 → C4 direction, see the red arrow in Figure 1) and the surface normal (*Z*-axis) are 60° or 120° for ethylbenzene, benzaldehyde, and aniline by assuming that the orientations of these molecules are similar to that of phenol.^46^ The sign of the angle depends on the molecular orientation and will be discussed below. Since the toluene molecule has a round shape and all the moieties are equally hydrophobic, we assume that the orientation of the interfacial toluene molecules is more randomized. In fact, the C–H symmetric stretching mode of the −CH_3_ group is much weaker for the toluene than for the ethylbenzene. From the experimentally obtained C–H stretching peak of these molecules, we estimated that the average orientation of the toluene is ∼71°.

## 4 Results and Discussion

### 4.1 Experimental Data

To understand the molecular orientations of the benzene derivatives, we first measured the HD-SFG spectra of the C–H stretching modes of the aliphatic −CH_3_ groups of toluene and ethylbenzene, the aliphatic C–H stretching mode of benzaldehyde, and the N–H stretching mode of aniline. The aliphatic C–H stretching mode is known to be dominated by the dipole contribution^47^ and thus is sensitive to the molecular orientation;^48^ a negative (positive) Im(χ_*YYZ*_^(2)^) signal for the aliphatic symmetric C–H stretching mode indicates the C → H bond pointing *up* to the air (*down* to the bulk). On the other hand, through the analogy of the O → H, a negative (positive) Im(χ_*YYZ*_^(2)^) signal for the N–H stretching mode indicates the N → H bond pointing *down* to the bulk (*up* to the air). As such, from the Im(χ_*YYZ*_^(2)^) signals for the C–H and N–H stretching modes, one can clearly understand the molecular orientations and thus the C1 → C4 direction.

**Figure 3:**
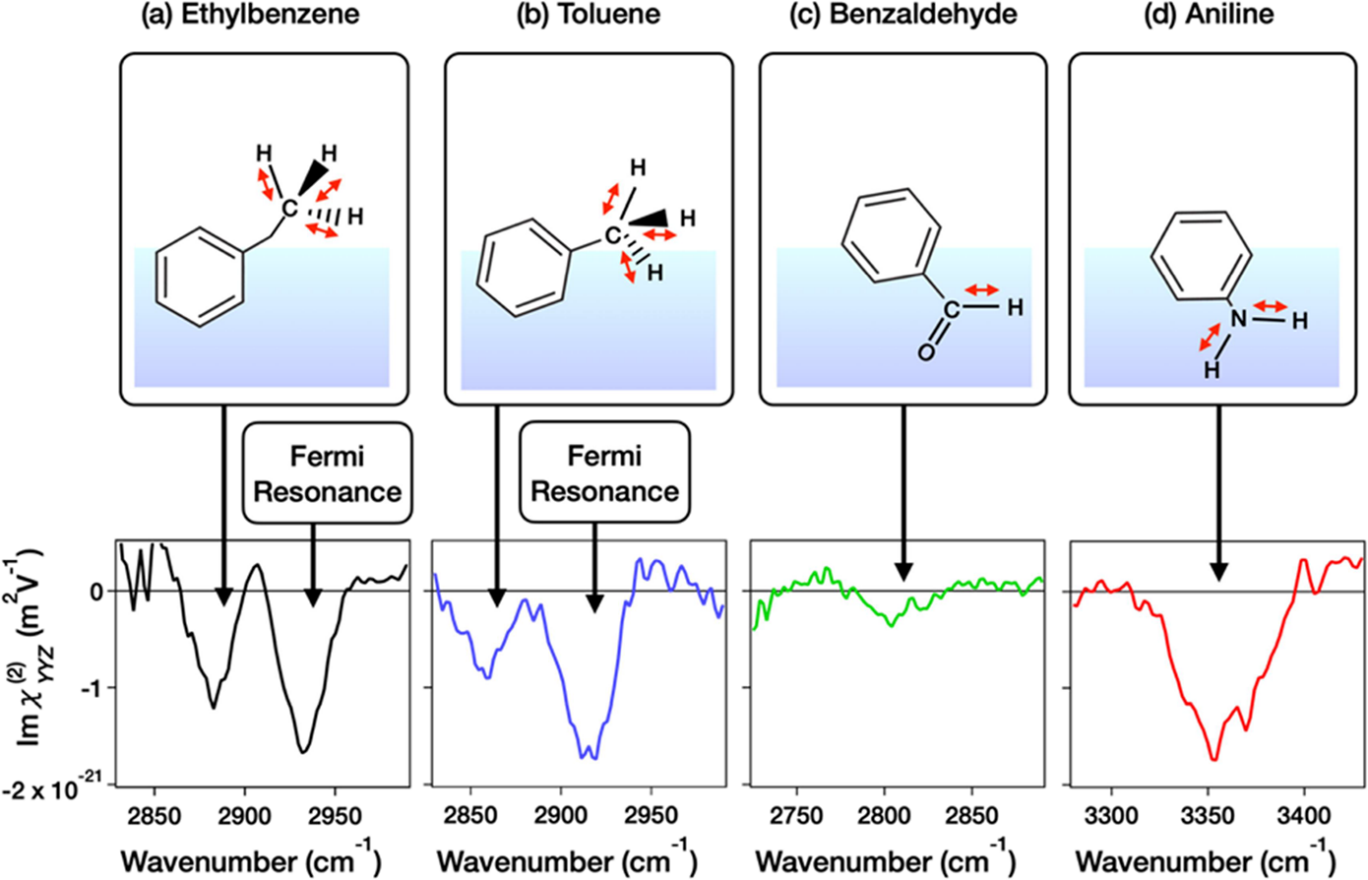
Im(χ_*YYZ*_^(2)^) spectra of (a) ethylbenzene in the 2825–3000 cm^–1^ region, (b) toluene in the 2825–3000 cm^–1^ region, (c) benzaldehyde in the 2725–2900 cm^–1^ region, and (d) aniline in the 3250–3450 cm^–1^ region. The schematics of the corresponding molecular vibrations and orientations are also shown as red arrows in the figure.

The measured Im(χ_*YYZ*_^(2)^) data are shown in Figure 3. The Im(χ_*YYZ*_^(2)^) spectra at the air/ethylbenzene and air/toluene interfaces in Figure 3a,b show the negative symmetric-stretching mode peak at 2880 and 2870 cm^–1^, demonstrating that both the −CH_3_ group of toluene and −C_2_H_5_ group of the ethylbenzene point *up* to the air and thus their C1 → C4 direction points *down* to the bulk.^22,49^ We assigned the negative peaks at 2930 and 2910 cm^–1^ in Figure 3a,b to the Fermi resonance. Furthermore, we assigned the positive peak at 2950 cm^–1^ in Figure 3b to the asymmetric C–H stretching mode of the CH_3_ group.^49^Figure 3c depicts the Im(χ_*YYZ*_^(2)^) spectra of air/benzaldehyde. A C–H stretching peak at 2820 cm^–1^ is negligibly small, manifesting that the C → H bond is almost parallel to the surface.^47,50,51^ Given that the oxygen atom of the aldehyde group is hydrophilic and thus tends to point *down* to the bulk,^47^ we concluded that the C1 → C4 direction points *up* to the air as displayed in Figure 3c. A negative peak at 3350 cm^–1^ in Im(χ_*YYZ*_^(2)^) spectra of air/aniline interface (Figure 3d) arises from the symmetric stretching mode of NH_2_ group.^52^ The negative sign of the peak indicates that the N → H group points *down* to the bulk and thus the C1 → C4 direction points *up* to the air.

**Figure 4:**
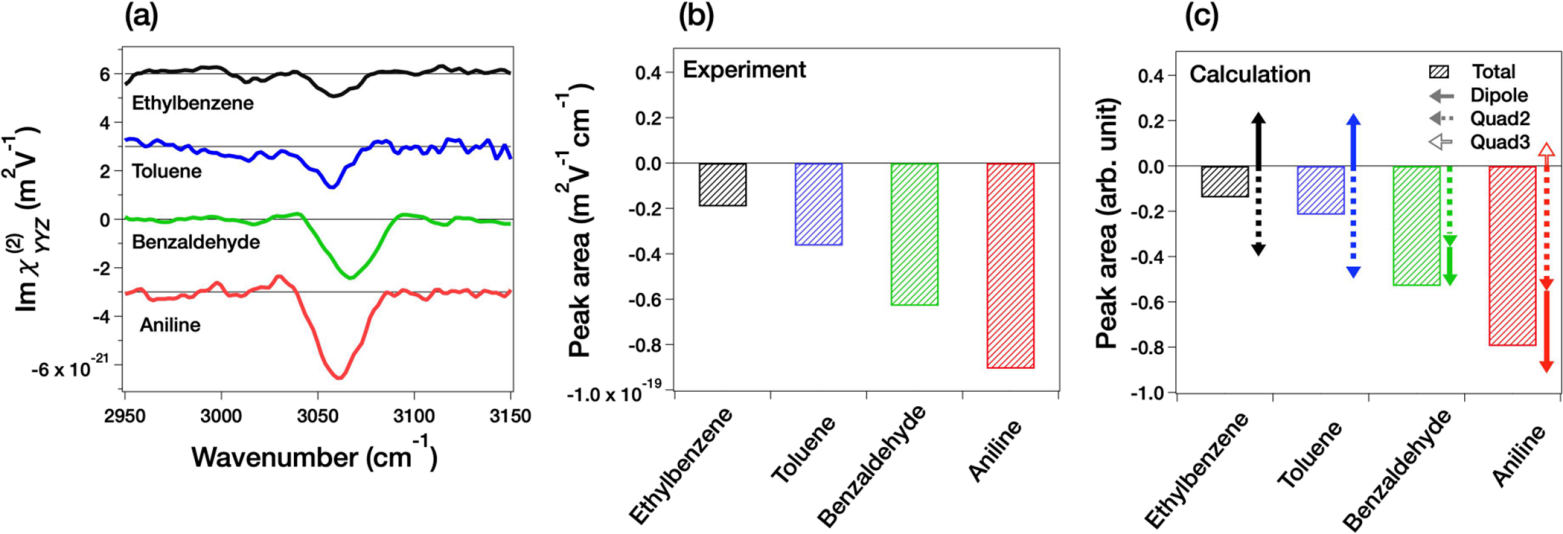
(a) Im(χ_*YYZ*_^(2)^) spectra of air/liquid benzene derivatives: ethylbenzene, toluene, benzaldehyde, and aniline in the 2950–3150 cm^–1^ region. These spectra are offset with 3.0 × 10^–21^ m^2^/V for clarity. (b) Experimentally obtained and (c) computed aromatic C–H stretching peak areas. In (c), the solid arrow, dotted arrow, outlined arrow, and shaded bar denote the dipole contribution, Quad2 contribution, Quad3 contribution, and sum of them, respectively. Note that Quad3 contributions are not shown for ethylbenzene, toluene, and benzaldehyde since their magnitudes are negligibly small.

After understanding the molecular orientations, we examine the relation of the molecular orientation vs the sign of the aromatic C–H stretch peak at ∼3060 cm^–1^. Figure 4a shows the Im(χ_*YYZ*_^(2)^) of air/benzene derivatives interface in the 2900–3200 cm^–1^ region. Surprisingly, we found that the aromatic C–H peaks are all negative, which starkly contrasts the variety of the molecular orientation of the studied benzene derivatives. Note that the negative ∼3050–3100 cm^–1^ Im(χ_*YYZ*_^(2)^) peak appears not only for the pure organic solvents of the benzene derivatives but also for benzene derivatives in water^9,10,30,53^ and proteins in water,^28,29,31,32^ indicating that the negative peak is rather universal, independent of the orientation of molecules. Our result clearly suggests a large quadrupole contribution in the aromatic C–H stretching mode.

Although the signs of the peak are all negative for these samples, the peak areas differ substantially. In fact, the areas of the aromatic C–H stretching peak summarized in Figure 4b show that the peak areas differ over a factor of three. To explore the origin of the drastic difference in the peak areas, we computed the quadrupole and the dipole contributions of aromatic C–H stretching mode.

### 4.2 Estimation of Dipole vs Quadrupole Contributions

To study the variation of peak areas in the Im(χ_*YYZ*_^(2)^) spectra, we performed the DFT calculation of these molecules. The result of the computations is shown in Figure 4c. The trend of the simulated area agrees well with the experimental data shown in Figure 4b. Based on the good agreement, we decomposed the contribution of the Im(χ_*YYZ*_^(2)^) peak area into those of Im(χ_*YYZ*_^(2), R, dipole^), Im(χ_*YYZ*_^(2), Quad2^), and Im(χ_*YYZ*_^(2), Quad3^). The dipole, Quad2, and Quad3 contributions are displayed in the solid, dotted, and outlined arrows in Figure 4c, respectively, while the Quad3 contribution is negligibly small and thus is not displayed for ethylbenzene, toluene, and benzaldehyde. The calculated Quad2 contribution is larger than the dipole contribution for all the studied benzene derivatives, making the aromatic C–H peak negative, irrespective of the molecular orientations of the studied molecules. The largest Quad2 contribution indicates that the aromatic C–H peak of the benzene derivatives in the SFG spectra ensures the presence of the organic molecules at the interfaces, while the negative sign of the aromatic C–H stretching peak in Im(χ_*YYZ*_^(2)^) does not necessarily reflect the orientation of the molecules. The conclusion that the largest contribution originates from Quad2 is similar to that drawn in a previous HD-SFG study of the benzene molecule.^24,26^

The Quad2 contribution is the strongest, but it is not totally dominant because the dipole contribution is not negligibly small compared with the Quad2 contribution. This non-negligible dipole contribution gives rise to the large variation of the aromatic C–H Im(χ_*YYZ*_^(2)^) peak areas. This observation indicates that we may be able to estimate the molecular orientation from the Im(χ_*YYZ*_^(2)^) aromatic C–H stretching data. In fact, the benzene derivatives with the C1 → C4 direction *down* to the bulk tend to give a smaller negative C–H aromatic peak, while those with the C1 → C4 direction *up* to the air tend to give a larger negative C–H aromatic peak in the Im(χ_*YYZ*_^(2)^) spectra.

Here, we consider a criterion to estimate the absolute orientation of the (bio-)molecules containing aromatic moieties. The sum of the dipole contributions of these four molecules is almost zero because of the similar magnitudes of positive and negative contributions (Figure 4c). Therefore, it is convenient to set the threshold by taking an average for these aromatic C–H peak area obtained experimentally. The Quad2 contribution of the aromatic C–H stretching mode in the Im(χ_*YYZ*_^(2)^) spectra can be estimated as
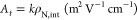
7where *k* is the averaged peak area normalized by the interfacial number densities of the benzene derivatives mentioned above and calculated to be
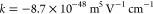
8ρ_N, int_ denotes the number density of an aromatic group of interest at the interface in units of m^–3^. If the peak area of the aromatic C–H group, *A*, is smaller than *A_t_* (*A* < *A_t_*), the C1 → C4 direction points *up* to the air since the dipole and quadrupole contributions interfere destructively. If *A* > *A_t_*, the interference must be constructive, indicating that the C1 → C4 direction points *down* to the bulk.

**Figure 5:**
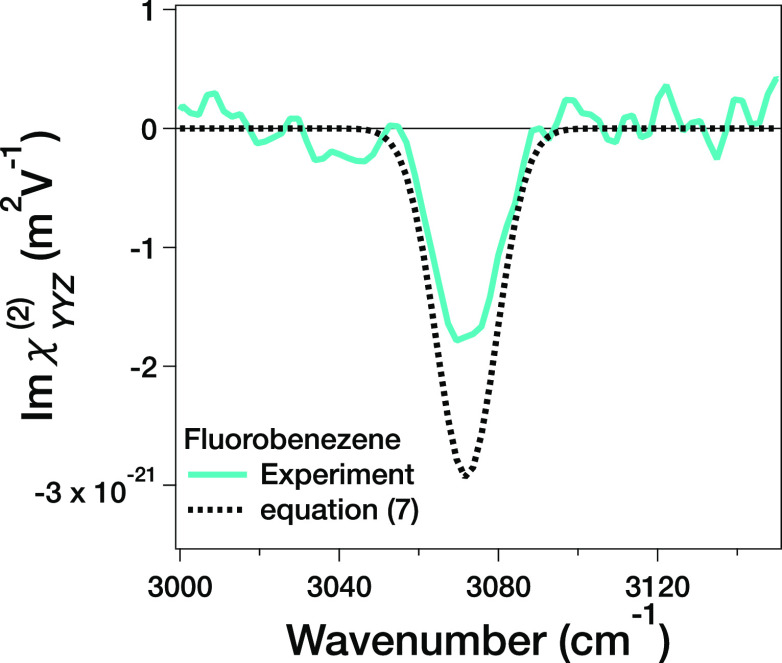
Im(χ_*YYZ*_^(2)^) spectra of the air/fluorobenzene interface. The solid line indicates the experimental data, and the dotted line is the lineshape estimated from eq 7 on the assumption that the bandwidth and the center frequency are the same as that of experimental spectra.

To examine whether the estimation of *A_t_* can be used for judging the molecular orientation of the benzene derivative, we measured the Im(χ_*YYZ*_^(2)^) spectrum of the air/fluorobenzene interface. The Im(χ_*YYZ*_^(2)^) spectrum is displayed in Figure 5. The obtained peak area is *A* = – 3.4 × 10^–20^ (m^2^ V^–1^ cm^–1^) and the threshold value of the Quad2 contribution, *A_t_*, was *A_t_* = – 5.6 × 10^–20^ (m^2^ V^–1^ cm^–1^) via eq 7. The estimated spectrum from eq 7 is shown as the dotted line in Figure 5. Because *A* > *A_t_*, one can expect that the C1 → C4 direction points *down* to the bulk.

To see whether the C1 → C4 direction pointing *down* makes sense, we carried out DFT calculation for computing energy of the two T-shaped^54^ fluorobenzene dimers; one is the dimer conformation where the perpendicular fluorobenzene has the C1 → C4 direction pointing *up*, while the other has the C1 → C4 direction pointing *down* (see Supporting Information). The conformation with the C1 → C4 direction pointing *down* is more stable by ∼2.8 *kT* than with the C1 → C4 direction pointing *up*. This implies that the fluorine atom tends to be up-oriented and the C1 → C4 direction pointing *down* at the air/fluorobenzene interface, consistent with the estimation using eq 7. This agreement between the theory and estimation using the experimental data indicates that the threshold value can be used for estimating the molecular orientation. Note that it is very challenging to observe the C–F stretching frequency of 1300 cm^–1^ with the heterodyne detection technique because the *y*-cut quartz we used for the LO generation reduces the 1300 cm^–1^ IR beam intensity drastically. Although an alternative of the *y*-cut quartz for LO generator was reported to facilitate HD-SFG down to ∼1000 cm^–1^,^55^ we decided to obtain the preferable orientation of the fluorobenzene through the computation.

## 5 Conclusions

We examined the origin of the aromatic C–H stretching peak in HD-SFG spectra by combining the experiment and DFT calculation of several benzene derivatives. Although the molecules investigated in this study showed different orientations at the interface, the signs of the aromatic C–H peaks were all negative. With the aid of the DFT calculation, we found that the interfacial quadrupole contribution shows the largest contribution. This is surprising because these benzene derivatives do not possess molecular symmetry, unlike benzene. However, we also revealed that the minor dipole contribution induces a considerable variation in the peak areas. Based on our observation, we suggest a simple criterion to estimate the molecular orientation by the peak area of the aromatic C–H stretching mode. Our finding refines the interpretation of the aromatic C–H peak in SFG spectra, providing fundamental insight into the SFG study of aromatic groups at the interface.

## Data Availability

All data required to evaluate the conclusions in the manuscript are available in the main text or the Supplementary Materials.

## Supporting Information Available

Calculation of *N*_int_, details of Fresnel factor removal, and estimation of the molecular orientation of fluorobenzene (PDF)
