# Supplementary material for: Does the Sum-Frequency Generation Signal of Aromatic C–H Vibrations Reflect Molecular Orientation?
Source: J Phys Chem B. 2023 Jun 7;127(23):5288–94. doi: 10.1021/acs.jpcb.3c01225 (PMC10278126; doi:10.1021/acs.jpcb.3c01225)
Supplement: Supplementary file 1 — jp3c01225_si_001.pdf [file jp3c01225_si_001.pdf]

Supporting Information for: Does the Sum-Frequency Generation Signal of Aromatic C-H Vibrations Reflect Molecular Orientation?

Fumiki Matsumura[1], Chun-Chieh Yu[1], Xiaoqing Yu[1], Kuo-Yang Chiang[1], Takakazu Seki[1,2], Mischa Bonn[1*], and Yuki Nagata[1*]

[1] Max Planck Institute for Polymer Research; Ackermannweg 10, 55128, Mainz, Germany.

[2] Graduate School of Science and Technology, Hirosaki University, Hirosaki, 036-8561, Aomori, Japan.

*Corresponding author. Email: bonn@mpip-mainz.mpg.de, nagata@mpip-mainz.mpg.de

Calculation of N_{int}

In equation (2) and (5), we computed N_{int} through:

$$\rho_{\mathrm{int}} \approx \frac{N_{\mathrm{int}}}{S \cdot Z_{\mathrm{int}}}, \tag{S1}$$

where ρ_{int} denotes the number density of molecules at the interfacial region. Here, we assumed $\rho_{\mathrm{int}} \approx \rho_{\mathrm{bulk}}$, where ρ_{bulk} denotes the number density of molecules in the bulk region. The average density in the SFG active interfacial region at the liquid/air interface is rather high; for example, at the water/air interface, the study shows that even the free O-H species of the topmost water experiences the average density of $\rho_{\mathrm{int}} \approx 0.7\rho_{\mathrm{bulk}}$ and the hydrogen-bonded O-H species experiences the density of $\rho_{\mathrm{int}} \approx \rho_{\mathrm{bulk}}$.[1,2] Since the position of the transition dipole moment is located at nearly the center of the molecule, the assumption of $\rho_{\mathrm{int}} \approx \rho_{\mathrm{bulk}}$ is reasonable. Furthermore, this assumption is consistent with our dielectric constant computed with the fully solvated (Lorentz) model.

Fresnel factor removal

We removed the Fresnel factor from the experimental result by using the relation:

$$(\chi^{(2)}_{\mathrm{eff}})_{ssp} = L_{YY}(\Omega_{\mathrm{SFG}})L_{YY}(\Omega_{\mathrm{Vis}})L_{ZZ}(\Omega_{\mathrm{IR}})\sin\theta_i(\Omega_{\mathrm{IR}})\chi^{(2)}_{YYZ}, \tag{S1}$$

where $L_{ii}(\Omega)(i = X,Y,Z)$denotes the Fresnel factor:[3]

$$L_{YY}(\Omega) = \frac{2\cos\theta_i(\Omega)}{\cos\theta_i(\Omega) + n_{\mathrm{bulk}}(\Omega)\cos\theta_t(\Omega)}, \tag{S2}$$

$$L_{ZZ}(\Omega) = \frac{2n_{\mathrm{bulk}}(\Omega)\cos\theta_i(\Omega)}{\cos\theta_t(\Omega) + n_{\mathrm{bulk}}(\Omega)\cos\theta_i(\Omega)}\frac{1}{n_{\mathrm{int}}^2(\Omega)}, \tag{S3}$$

at the frequency of Ω, and θ_i (θ_t) is the incident (reflected) angle. n_{bulk} and n_{int} denote the refractive index in the bulk and at the interface. We used the fully solvated (Lorentz) model to calculate the refractive index at the interface.[1,2]

In Table S1, we summarized the refractive indexes used in the Fresnel factor removal. We assumed the refractive indexes are constant from the SFG to the visible frequency region. Since the refractive indexes of aniline, benzaldehyde, and ethylbenzene in the IR frequency region are not available in the literature, we estimated these values on the assumption that the dispersion of the refractive index from the visible to the IR frequency region is similar to that of toluene molecule.

Table S1. Refractive indexes used in the Fresnel factor removal. Estimated values are shown with *.

	SFG	Vis	IR
Aniline	1.59[4]	1.59[4]	1.56*
Benzaldehyde	1.54[5]	1.54[5]	1.51*
Toluene	1.50[6]	1.50[6]	1.47[7]
Ethylbenzene	1.50[8]	1.50[8]	1.47*
Fluorobenzene	1.47[6]	1.47[6]	1.44[9]

Estimation of the molecular orientation of fluorobenzene

For simplicity, we consider a benzene derivative molecule located at the interface parallel to the surface. For such a benzene derivative, one can consider the intermolecular interactions depicted in the left panel of Figure S1; one is the T-shaped conformation (the right panel of Figure S1, structure 3), while the other is the parallel conformation (the middle panel of Figure S1 structures 1, 2, and other structures are discussed in the literature[10]). In the parallel conformations of the dimer, the interactions are the same irrespective of the direction of the benzene derivatives. Thus,

only the T-shape conformation gives rise to the different energy based on the different orientations. As such, we consider the conformational energy of the T-shaped dimer and then estimate the preferable orientation of the benzene derivatives.

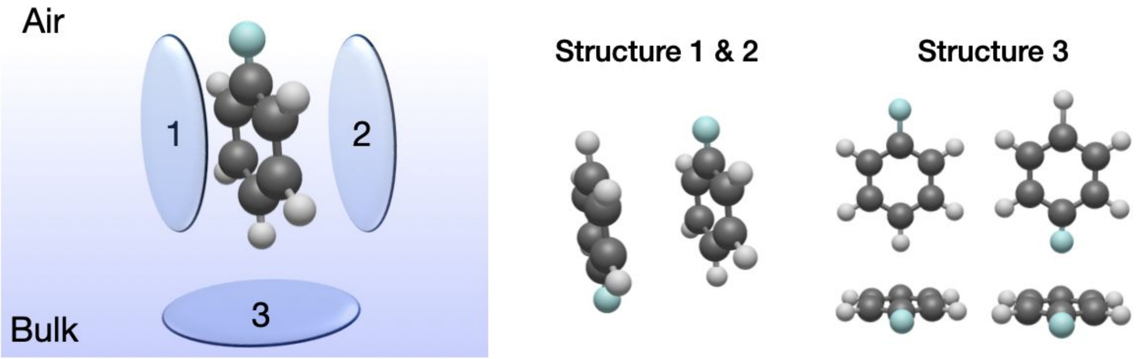

Figure S1. Left panel: Possible molecular conformations near the benzene derivative molecule at the liquid-air interface. The transparent circles indicate the possible positions of counter molecules. Middle panel: Dimer conformation when the counter molecules are located at positions 1 & 2. Right panel: Dimer conformation when the counter molecules are located at position 3.

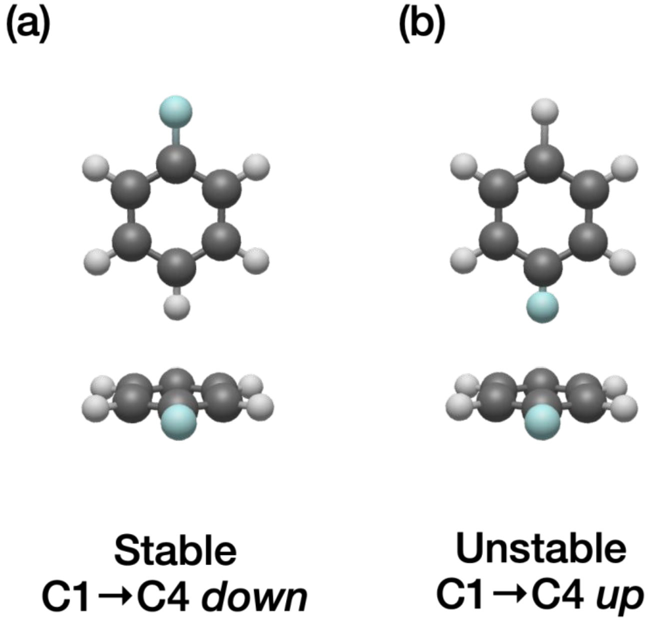

Figure S2. The conformations of the fluorobenzene dimers. (a) the perpendicular fluorobenzene has the C1→C4 direction pointing *down*, (b) the perpendicular fluorobenzene has the C1→C4 direction pointing *up*.

References

(1) Chiang, K.-Y.; Seki, T.; Yu, C.-C.; Ohto, T.; Hunger, J.; Bonn, M.; Nagata, Y. The Dielectric Function Profile across the Water Interface through Surface-Specific Vibrational Spectroscopy and Simulations. *Proc. Natl. Acad. Sci.* **2022**, *119*, e2204156119.

(2) Yu, X.; Chiang, K.-Y.; Yu, C.-C.; Bonn, M.; Nagata, Y. On the Fresnel Factor Correction of Sum-Frequency Generation Spectra of Interfacial Water. *J. Chem. Phys.* **2023**, *158*, 044701.

(3) Akihiro Morita. *Theory of Sum Frequency Generation Spectroscopy*; Springer Singapore, 2018.

(4) Kumar, S.; Jeevanandham, P. Densities, Viscosities, Refractive Indices and Excess Properties of Aniline and o-Anisidine with 2-Alkoxyethanols at 303.15K. *J. Mol. Liq.* **2012**, *174*, 34–41.

(5) Valiskó, M.; Boda, D.; Liszi, J.; Szalai, I. Relative Permittivity of Dipolar Liquids and Their Mixtures. Comparison of Theory and Experiment. *Phys. Chem. Chem. Phys.* **2001**, *3*, 2995–3000.

(6) Wohlfarth, C.; Wohlfarth, B. *Refractive Indices of Inorganic, Organometallic, and Organononmetallic Liquids, and Binary Liquid Mixtures*; Landolt-Börnstein - Group III Condensed Matter; Springer-Verlag Berlin Heidelberg New York, 1996; Vol. 38A.

(7) Bertie, J. E.; Jones, R. N.; Apelblat, Y.; Keefe, C. D. Infrared Intensities of Liquids XIII: Accurate Optical Constants and Molar Absorption Coefficients Between 6500 and 435 cm^{-1} of Toluene at 25°C, from Spectra Recorded in Several Laboratories. *Appl. Spectrosc.* **1994**, *48*, 127–143.

(8) Al-Kandary, J. A.; Al-Jimaz, A. S.; Abdul-Latif, A.-H. M. Densities, Viscosities, and Refractive Indices of Binary Mixtures of Anisole with Benzene, Methylbenzene, Ethylbenzene, Propylbenzene, and Butylbenzene at (293.15 and 303.15) K. *J. Chem. Eng. Data* **2006**, *51*, 99–103.

(9) Keefe, C. D.; Barrett, J.; Jessome, L. L. Optical Constants and Vibrational Assignment of Fluorobenzene between 4000 and 400cm^{-1} at 25°C. *J. Mol. Struct.* **2005**, *734*, 67–75.

(10) Pitoňák, M.; Neogrády, P.; Řezáč, J.; Jurečka, P.; Urban, M.; Hobza, P. Benzene Dimer: High-Level Wave Function and Density Functional Theory Calculations. *J. Chem. Theory Comput.* **2008**, *4*, 1829–1834.
